# Protective Effect of the *Abelmoschus manihot* Flower Extract on DSS-Induced Ulcerative Colitis in Mice

**DOI:** 10.1155/2021/7422792

**Published:** 2021-08-09

**Authors:** Bensheng Wu, Qing Zhou, Zongqi He, Xiaopeng Wang, Xueliang Sun, Yugen Chen

**Affiliations:** ^1^Department of Colorectal Surgery, Affiliated Hospital of Nanjing University of Chinese Medicine, Nanjing, Jiangsu 210029, China; ^2^Department of Colorectal Surgery, Suzhou Hospital Affiliated with Nanjing University of Chinese Medicine, Suzhou 215009, China

## Abstract

**Background:**

The flower of *Abelmoschus manihot* (AM) has been widely used in the treatment of chronic inflammatory diseases, including ulcerative colitis. This paper aimed to confirm the therapeutic effect of AM on ulcerative colitis (UC) and explore its mechanism.

**Methods:**

Mouse models were induced by 2.5% dextran sulfate sodium (DSS) and treated with AM. UC signs, symptoms, colon macroscopic lesion scores, and disease activity index (DAI) scores were observed. Colon levels of interleukin- (IL-) 6, IL-1*β*, IL-18, IL-17, tumor necrosis factor- (TNF-) *α*, and IL-10 were quantified by ELISA. The colon protein expression levels of NLRP3, ASC, caspase 1 p10, *β*-arrestin1, ZO-1, occludin-1, and claudin-1 were examined by immunohistochemistry and western blotting. The mRNA levels of IL-1*β*, IL-18, NLRP3, ASC, and caspase 1 p10 in the colon were determined by real-time quantitative polymerase chain reaction (qPCR).

**Results:**

After treatment with AM, the mortality of mice, pathological damage to the colon, splenomegaly, and the spleen coefficient were decreased. AM reduced the levels of proinflammatory cytokines (IL-6, IL-1*β*, IL-18, IL-17, and TNF-*α*) and increased the level of IL-10. The mRNA expression levels of NLRP3, ASC, and caspase 1 in colon tissue were decreased by AM in a dose-dependent manner. In addition, AM also reduced the protein expression of NLRP3, ASC, caspase 1 p10, IL-1*β*, IL-18, and *β*-arrestin1 in the colon tissue of model mice. Western blot analysis confirmed that AM increased the expression of occludin-1, claudin-1, and ZO-1 in a dose-dependent manner.

**Conclusion:**

This study shows that AM has a significant therapeutic effect on mice with UC, and the mechanism may be related to the inhibition of the *β*-arrestin1/NLRP3 inflammasome signaling pathway and the protection of intestinal barrier function.

## 1. Introduction

Ulcerative colitis (UC) is a common chronic intestinal inflammatory disease and is a difficult digestive system disease [1, 2]. The clinical symptoms of the patients are often fatigue, increased frequency of bowel movements, mucus discharge, nighttime bowel movements, and abdominal discomfort [1, 3]. UC has a long course and is prone to recurrence, which increases the risk of colon cancer [4].

The specific pathogenesis of UC has not been fully elucidated. People with genetic susceptibility to UC exhibit activated immune systems due to antigen stimulation, the interactions of various environmental factors, and a series of abnormal immune responses, thereby causing intestinal inflammation, and the dysregulation of immune function plays an important role in the development of UC [1]. The main drugs currently used for UC management include 5-ASA, immunosuppressants, and anti-TNF-*α* drugs, which only help alleviate disease symptoms, and these drugs may induce adverse reactions and side effects [5, 6]. Therefore, identifying for drugs with good effects and high safety is our current research goal.

The innate immune system of the intestinal tract is the body's first line of defense against various bacterial antigens. The weakened innate immune system of UC patients causes the accumulation of bacterial antigens and stimulates a cascade inflammatory response in the acquired immune system. NLRP3 inflammasomes play a certain role connecting the immune system and intestinal bacteria and can maintain intestinal homeostasis. NLRP3 inflammasome is the most studied NLR family member that composed of NLRP3, apoptosis-associated speck-like protein containing a caspase recruitment domain (ASC) and procaspase 1 [7]. Inflammasome activation causes the automatic cleavage of caspase 1, thereby mediating the maturation of IL-1*β* and IL-18 [8]. Among of the many inflammasomes, the NLRP3 inflammasome is activated by a variety of stimuli, such as bacterial toxins, damage-associated molecular patterns (DAMPs), and pathogen-associated molecular patterns (PAMPs) [9]. The NLRP3 inflammasome has a proinflammatory effect during the onset of colitis. Treatment with the NLRP3-dependent procaspase 1, IL-1*β,* and the IL-18 inhibitor Fclla-2 can reduce colon inflammation in mice [10], and the NLRP3 inflammasome can initiate and promote the inflammatory response in colitis [11].

Intestinal barrier dysfunction also plays a key role in the pathogenesis of IBD [12]. Studies have confirmed that the intestinal barrier function of mice with UC is impaired and that the expression of proteins, such as ZO-1 and claudin-1, which maintain the tight junction structure in the barrier, is reduced [13, 14]. Tight junctions are closely related to the intestinal barrier function of UC. Therefore, it may be important to develop therapeutic drugs to treat UC by inhibiting the intestinal NLRP3 inflammasome and protecting the integrity of the intestinal barrier.

The flower of *Abelmoschus manihot* (Chinese name: Huang-Shu-Kui Hua) is a kind of traditional medicine. AM was first recorded in Jiayou Materia Medica, which is widely distributed and rich in resources. AM has been widely used in the clinic, and current pharmacological studies have shown that AM has excellent anti-inflammatory and antioxidant properties [15, 16]. Our research group has confirmed the role of AM in inflammatory bowel disease [17, 18]. The traditional Chinese medicine compound preparation (Huangkui Lianchang Decoction) has a significant effect on UC model mice [19], but the specific mechanism of AM as the main drug has not been further studied. Therefore, we hypothesize that AM can inhibit the NLRP3 inflammasome and protect intestinal barrier function to treat UC.

## 2. Materials and Methods

### 2.1. Experimental Animals

Male C57/BL6 mice (6 weeks old, 20 ± 2 g) were obtained from SPF (Beijing) Biotechnology Co., Ltd. The laboratory animal production license number is SCXK (JING): 2016-0002. Experimental protocols were in accordance with National Institutes of Health regulations and approved by the Institutional Animal Care and Use Committee. All mice were fed adaptively for seven days and were housed under standard SPF rooms and received standard food and sterile water ad libitum at a constant temperature (20–25°C) and humidity (65–70%) with a 12-hour light/dark cycle. The experimental protocol was approved by the Animal Ethics Committee of the Suzhou Affiliated Hospital of Nanjing University of Traditional Chinese Medicine.

### 2.2. Drugs and Reagents

#### 2.2.1. Preparation of *Abelmoschus manihot*

AM was provided by the Pharmaceutical Department of Jiangsu Provincial Hospital of Traditional Chinese Medicine (purchased from Anhui Concord Co., Ltd. (batch number 19081010)). The specimen was stored at the Herbarium of Nanjing University of Chinese Medicine for future reference and verification. Based on the method established by Professor Guo Jianming of Nanjing University of Chinese Medicine (Nanjing) [20], we used a grinder to crush the AM and then used a 100-mesh sieve to filter and mix it thoroughly. The dried pollen was extracted twice with 80% ethanol under reflux (70°C) for 2 h each time. After filtering and combining the filtrate, it was concentrated to dryness under vacuum at 60°C on a rotary evaporator. The extract was then weighed and dissolved in distilled water before use.

#### 2.2.2. Reagents and Antibodies

Dextran sodium sulfate (DSS) was obtained from MP Biomedicals (MW; 36000–50000, MP Biomedicals, USA). Absolute ethanol and xylene (Chemical Reagent, Sinopharm Group, China) were purchased. Enzyme-linked immunosorbent assay (ELISA) kits for IL-6 (#CSB-E04639m), IL-1*β* (#CSB-E08054m), IL-18 (#CSB-E04609m), IL-17 (#CSB-E04608m), TNF-*α* (#CSB-E04741m), and IL-10 (#CSBE04594m) were purchased from Cusabio Biotech Co. (Wuhan, Hubei, China). Anti-NLRP3 (#AG-20B-0014-C100, Adipogen, USA), Anti-ASC (#SC-22514R, Santa Cruz Biotechnology, USA), anticaspase 1 p10 (#SC-22166, Santa Cruz Biotechnology, USA), anti-IL-1*β* (#12242, Cell Signaling, USA), anti-IL-18 (#ab68435, Abcam, USA), anti-ZO-1 (#sc-33725; Santa Cruz Biotechnology, USA), antioccludin-1 (#71–1500, Invitrogen, USA), anticlaudin-1 (#51–9000, Invitrogen, USA), and anti-*β*-actin (#ab6276,Abcam, USA) antibodies were used. TRIzol reagent (Life Company, USA), DEPC water (Biyuntian Biotechnology, China), isopropanol (Chengdu Kelon Chemical, China), SYBR Premix Ex TaqTM, PrimeScript^TM^ RT master mix (#RR820A; Takara Bio, Inc), the hematoxylin-eosin staining kit (Cusabio Biotech, China), the DAB color reagent histochemistry kit (Cusabio Biotech, China), the BCA protein quantitative detection kit (#G2026, Cusabio Biotech, China), ECL (#G2014, Cusabio Biotech, China), and TBS buffer (#G0001-2L, Cusabio Biotech, China) were purchased.

### 2.3. DSS-Induced Colitis and Drug Treatment

**Figure 1:**
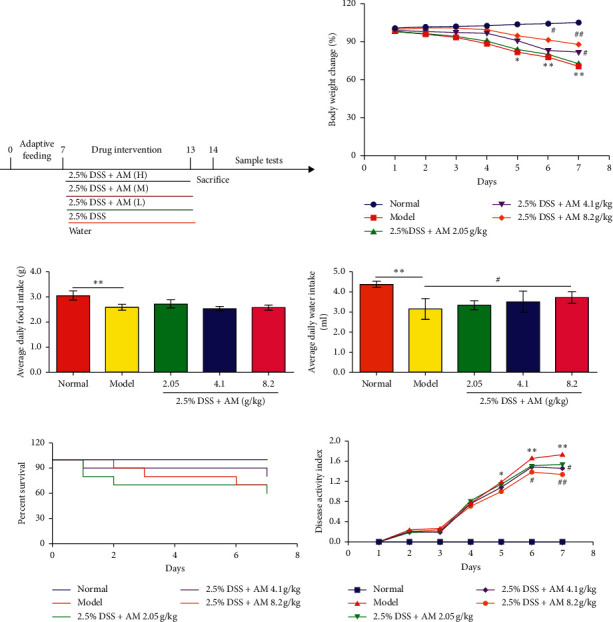
AM treatment alleviates DSS-induced colitis in mice. Mice were fed 2.5% DSS and orally administered AM (2.05, 4.1, and 8.2 g/kg) for 7 days. (a) Schematic diagram of this experiment. (b) Body weight changes. (c) Average daily water intake. (d) Average daily food intake. (e) Percent survival. (f) Disease active index. The data are shown as the mean ± SEM in each group. ^*∗*^*P* < 0.05. ^*∗∗*^*P* < 0.01 vs. the control group; ^#^*P* < 0.05. ^##^*P* < 0.01 vs. the DSS control group.

The administration of 2.5% DSS in drinking water for 7 days led to acute colitis (Figure 1(a)). Thirty mice were randomly divided into 5 groups, with 6 mice in each group: (1) normal control group, (2) DSS model group, (3) AM-L group, (4) AM-M group, and (5) AM-H group. The daily dose of AM for normal adults was 20 g/day, and the bioavailability in the AM-M group was 4.1 g/kg; doses 1/2 and 2 times the bioavailability of that in the AM-M group were used for the AM-L group (2.05 g/kg) and AM-H group (8.2 g/kg), respectively. Except for the normal control group, which was given distilled water, the other groups were only allowed to drink 2.5% DSS throughout the experiment. The management method of the mice in each group is shown in the schematic diagram (Figure 1(a)). Body weight, mouse food intake, and water intake were measured daily.

  Average daily food = total food intake weight/number of animals
  Average water intake = total water intake volume/number of animals

### 2.4. Observation of UC Signs, Symptoms, and Disease Activity Index (DAI) Scores

During the course of the experiment, the weights of mice in each group, food and drinking water consumption, mortality, stool consistency, and diarrhea were observed by two blinded individuals every day. The DAI was calculated accordingly, as shown in Table 1 [21].

### 2.5. Management of Colon Tissue and Assessment of Pathological Changes

According to the National Institutes of Health ARAC guidelines, carbon dioxide was used to euthanize the mice. Colon tissue was quickly isolated from the mice and photographed, and then the colon length was measured in a naturally flat state. The colon was then rinsed with PBS, placed on gauze to absorb water, and weighed. The colon was then divided into three equal parts. The proximal and middle segments were immediately stored in liquid nitrogen. The distal colon tissue was fixed with 4% paraformaldehyde, embedded in paraffin, and cut into 4 *μ*m sections. The sections were stained with hematoxylin and eosin, followed by microscopic observation, and histological scores were assessed in a blinded manner. The histological score was calculated accordingly, as shown in Table 2 [22].

### 2.6. Examination of the Spleen Coefficient

Spleens were separated, photographed, and weighed after the mice were sacrificed, and then the organ coefficient was calculated. Spleen coefficient (%) = (organ weight/body weight on the 7th day) ×100%.

### 2.7. Immunohistochemistry Assay

The paraffin sections were deparaffinized in water, and the sections were placed in a citrate buffer repair box at high temperature for antigen retrieval, cooled naturally, and washed 3 times with PBS. After antigen retrieval, the primary antibody was added dropwise and incubated overnight at 4°C, and the secondary antibody was added dropwise and incubated at room temperature for 50 min. The sections were washed 3 times with PBS. DAB was added for color development, and the slides were dehydrated, make transparent, dried, and sealed with neutral gum. The protein expression of NLRP3, ASC, caspase 1 p10, *β*-arrestin1, Zo-1, occludin-1, and claudin-1 in colon tissue was observed and recorded under a microscope.

### 2.8. Inflammatory Cytokine Determination by the ELISA Assay

The amounts of IL-6, IL-1*β*, IL-18, IL-17, TNF-*α*, and IL-10 in colon tissues were quantified by ELISA kits according to the manufacturer's instructions.

### 2.9. RNA Extraction and Quantitative Real-Time PCR (qRT-PCR)

Trizol was used as the extraction reagent to extract total RNA from mouse colon tissues, and reverse transcription was performed according to the kit instructions. After reverse transcription, SYBR Green was used as a fluorescent marker and GAPDH was used as an internal reference control, and 1 ul cDNA was added to the 20 ul reaction system. Perform amplification. The amplification conditions were 95°C for 30 s, 1 cycle, 95°C for 5 s, 60°C 30 s, 40 cycles, 95°C for 15 s, 60°C 60 s, and 1 cycle. The 2^−ΔΔCt^ method was used to calculate the relative expression of mRNA. The primer sequences were as follow.

### 2.10. Western Blot Analysis

Proteins were extracted from mouse colon tissue, quantified, denatured, and then subjected to SDS-PAGE. After electrophoresis, the proteins were transferred to the membrane, and after the membrane was blocked, the primary antibody was added and incubated overnight at 4°C. After the membrane was washed with TBST, secondary antibody was added and incubated at room temperature for 30 min. Quantity One software (Bio-Rad Laboratories, Hercules, CA, USA) was used for densitometric analysis.

### 2.11. Statistical Analysis

Statistical analysis was performed using GraphPad Prism 6.0 software. The results are expressed as the mean ± SEM. Data were statistically assessed by one-way ANOVA followed by Dunnett's test for comparisons between the control group and multiple dose groups. The level of significance was set at a *P* value of 0.05.

## 3. Results

### 3.1. AM Can Relieve the Signs and Symptoms of DSS-Induced UC in Mice

DSS-induced colitis is one of the most widely used animal models to study the pathogenesis of IBD and evaluate therapeutic drugs. In this experiment, it was observed that 7 days of drinking a 2.5% DSS solution ad libitum resulted in a significant reduction in body weight in the mice (Figure 1(b)), decreased food and water intake (Figures 1(c) and 1(d)), and increased mortality (Figure 1(e)). The DAI increased compared to that in the normal group (Figure 1(f)). Most importantly, body weight began to decline rapidly on the fifth day, and the condition of the animals also deteriorated rapidly.

However, treatment significantly inhibited DSS-induced weight loss, and the weight gradually returned to higher levels in a dose-dependent manner (Figure 1(b)). Water intake increased significantly (Figure 1(c)), while the decrease in the DAI score (Figure 1(f)) was dose dependent. These data indicate that AM has a protective effect on DSS-induced colitis in mice.

### 3.2. AM Alleviates Colonic Damage in Mice with DSS-Induced Colitis

**Figure 2:**
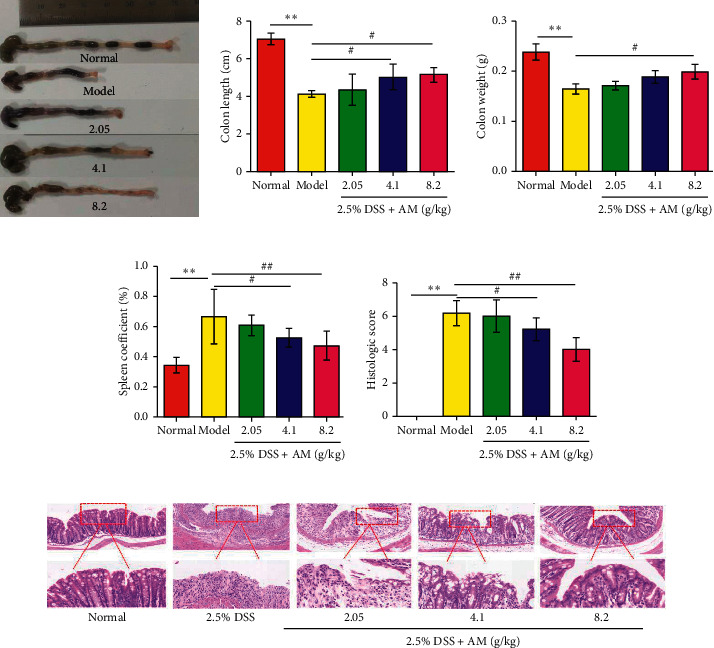
Effects of AM on colonic pathological changes in mice with DSS-induced colitis. (a) Representative images of the colon in each group. (b) Colon length. (c) Colon weight. (d) Spleen coefficient. (e) Histopathological score. (f) Representative images of hematoxylin and eosin (H&E) staining in colon tissues (magnification: ×200 and ×400). The data are shown as the mean ± SEM.

During the course of UC changes, a series of colonic pathological changes associated with inflammation occur [23]. Therefore, the effect of AM on the pathological changes in the colons in DSS-induced colitis in mice was further studied. As shown in Figure 2, the DSS control group had a much shorter colon length, reduced intestinal weight, and limited general morphology and histopathology scores compared to those of the normal group.

These results suggested that DSS administration caused significant colonic pathological changes, indicating that the UC mouse model was successfully stablished. However, treatment with AM could effectively alleviate colonic damage. Compared with that in the DSS control group, AM administration had a significant inhibitory effect on DSS-induced colon contracture (Figures 2(a) and 2(b)).

Compared with that in the DSS control group, AM reduced inflammation and restored intestinal weight. Furthermore, mice in the DSS control group developed extensive histopathological damage and colonic inflammatory infiltration. The intestinal morphological damage in DSS control mice, including edema, ulcers, and tissue necrosis, was significantly alleviated by AM (Figures 2(e) and 2(f)). Mice with DSS-induced colitis develop splenomegaly [24]. Immune system disorder leads to increased spleen size in mice with DSS-induced colitis. The spleen coefficient of the model group was significantly higher than that of the normal group. High-dose AM reduced the spleen coefficient and effectively inhibited spleen enlargement. AM could partially improve immune system disorders in mice with ulcerative colitis and effectively counteract spleen enlargement (Figure 2(d)). These data indicate that AM can protect the colon in DSS-induced colitis mice.

### 3.3. Effect of AM on the Expression of IL-6, IL-1*β*, IL-18, IL-17, TNF-*α,* and IL-10 in the Colons of DSS-Induced Mice

**Figure 3:**
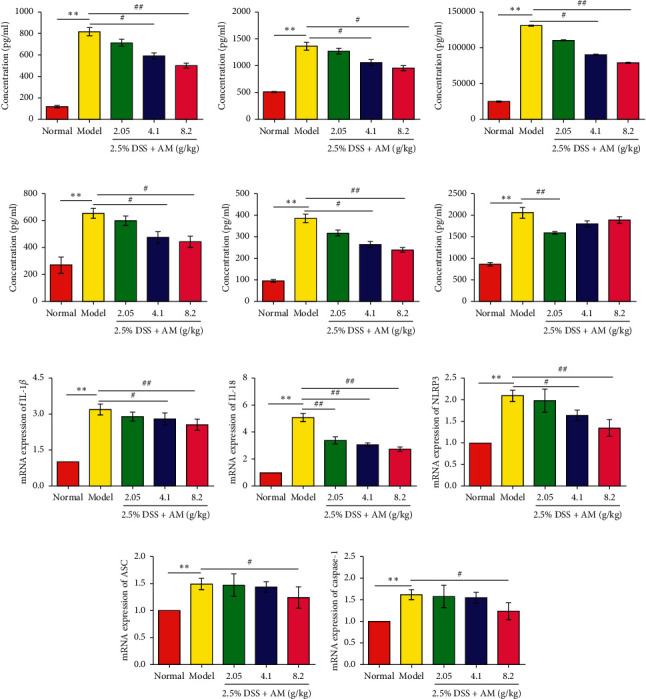
Effects of AM on cytokines and NLRP3 inflammasomes in colon tissue. (a) The expression of IL-6, (b) IL-1*β*, (c) IL-18, (d) IL-17, (e) TNF-*α*, and (f) IL-10. (g) The mRNA expression of IL-1*β*, (h) IL-18, (i) NLRP3, (j) ASC, and (k) caspase 1. The data are presented as the mean ± SEM. ^*∗∗*^*P* < 0.01, compared to the normal group; ^#^*P* < 0.05 and ^##^*P* < 0.01, compared to the model group.

Inflammatory cytokine expression plays an important role in the pathogenesis of IBD [25]. Therefore, we further studied the regulatory effect of AM on intestinal inflammation in mice with DSS-induced colitis. Compared with those in the normal control group, all cytokines in the DSS treatment group were significantly increased (Figure 3). AM reduced the levels of proinflammatory cytokines (IL-6, IL-1*β*, IL-18, IL-17, and TNF-*α*) (Figures 3(a)–3(e)) and increased the level of IL-10 (Figure 3(f)). Therefore, AM can inhibit the DSS-induced inflammatory response in UC mice by reducing the secretion of proinflammatory cytokines.

### 3.4. AM Suppresses Activation of the NLRP3 Inflammasome and *β*-Arrestin1 Signaling Pathway in Mice with DSS-Induced Colitis

**Figure 4:**
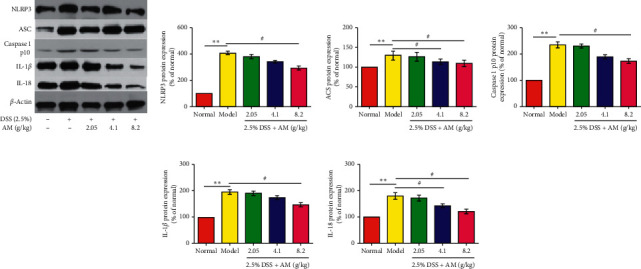
The effect of AM on the expression of the NLRP3 inflammasome and the *β*-arrestin1 signaling pathway in mice with DSS-induced colitis. The protein expression levels of NLRP3, ASC, caspase 1 p10, IL-1*β*, and IL-18 (a) in colonic tissue were measured by western blotting. The protein expression levels of NLRP3 (b), ASC (c), caspase 1 p10 (d), IL-1*β* (e), and IL-18 (f) were quantitated by Image software. The data are presented as the mean ± SEM. ^*∗∗*^*P* < 0.01, compared to the normal group; ^#^*P* < 0.05 and ^##^*P* < 0.01, compared to the model group.

**Figure 5:**
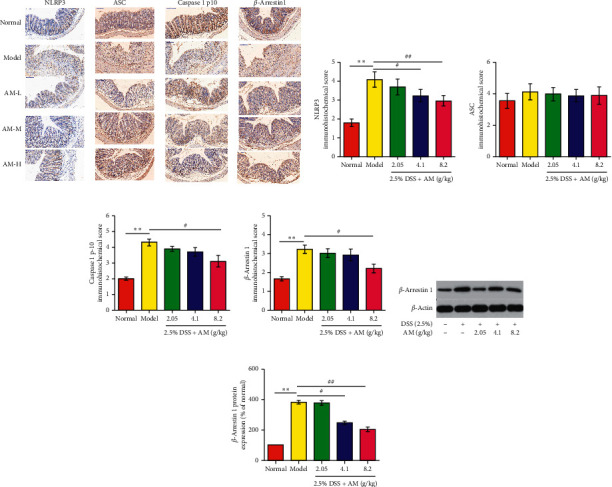
Effects of AM on the expression of NLRP3 inflammasome proteins and *β*-arrestin1 signaling proteins in the colon tissue of mice with DSS-induced colitis. (a) Immunohistochemical staining results of the NLRP3 inflammasome and *β-*arrestin1 in colon tissue (×100). (b–e) Score of the immunohistochemical staining results. The protein expression of *β*-arrestin1 (f). The protein expression of *β*-arrestin1 was quantitated by Image software (g). The data are presented as the mean ± SEM. ^*∗∗*^*P* < 0.01, compared to the normal group; ^#^*P* < 0.05 and ^##^*P* < 0.01, compared to the model group.

Current studies have confirmed that the production of IL-1*β* and IL-18 is closely related to the activation of the NLRP3 inflammasome. In these experiments, the secretion of IL-1*β* and IL-18 was significantly inhibited by AM. The mRNA expression of these factors was further examined by RT-PCR. The results suggested that the mRNA expression of IL-1*β* and IL-18 in the colon tissue of UC mice was significantly decreased (Figures 3(g) and 3(h)). Based on these results, we hypothesize that AM can prevent UC by inhibiting the NLRP3 inflammasome signaling pathway. The results showed that the mRNA expression levels of NLRP3, ASC, and caspase 1 in DSS-induced colon tissue were significantly higher than those in the normal control group and gradually decreased with increasing AM doses (Figures 3(i)–3(k)). The protein levels of NLRP3, ASC, caspase 1 p10, IL-1*β,* and IL-18 in the DSS treatment group were also significantly reduced (Figures 4(a)–4(f) and Figures 5(a)–5(d)). These findings suggest that AM can inhibit the NLRP3 inflammasome pathway, mainly by inhibiting the activation of NLRP3 inflammasomes.

Studies have confirmed that *β*-arrestin1, an important molecule in the *G* protein coupled receptor signaling pathway, plays a key role in the activation of NLRP3 inflammasomes [26]. *β*-arrestin1 can regulate the activation of the NLRP3 inflammasome and plays an important role in promoting the activation of the NLRP3 inflammasome [26]. Therefore, we further studied the effect of AM on the *β*-arrestin1/NLRP3 inflammatory signaling pathway. The results showed that AM significantly inhibited the protein expression of *β*-arrestin1 in colitis mice (Figures 5(a), 5(e), 5(f) and 5(g)). AM can regulate the expression of *β*-arrestin1 and block the activation of the NLRP3 signaling pathway, thereby inhibiting the activation of the proinflammatory factors IL-1*β* and IL-18 and reducing the inflammatory response.

### 3.5. AM Can Effectively Protect the Intestinal Barrier Function

Imbalance in the intestinal mucosal barrier is an early initiation event in UC. Tight junction proteins (occludin-1, claudin-1, and ZO-1) are essential for maintaining the integrity of the intestinal mucosal barrier [27]. Intestinal mucosal monolayer columnar epithelial cells are composed of tight junction proteins such as occludin and claudins and peripheral membrane proteins such as ZO-1. Claudins are transmembrane proteins that are the backbone of tight junctions and play an important role by restricting fluid flow. Through the cell alternative pathway, claudins and occludin participate in the transfer of macromolecular substances. As a peripheral membrane protein, ZO-1 plays a pivotal role in the distribution of tight junctions and the maintenance of intestinal mucosal permeability [28, 29]. The integrity of tight junction proteins is essential for maintaining the healing of the mucosa in UC.

**Figure 6:**
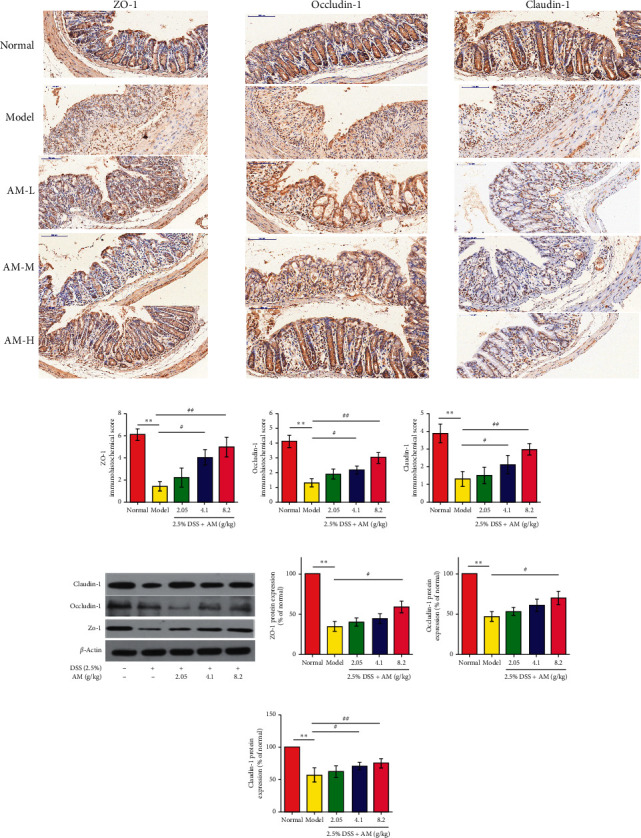
AM protects the intestinal barrier function of mice with DSS-induced colitis. Immunohistochemical staining results of ZO-1, occludin-1, and claudin-1 in colon tissue (×100) (a–d). The protein expression of claudin-1, occludin-1, and ZO-1 (e). The protein expression of ZO-1, occludin-1, and claudin-1 was quantitated by Image software (f–h). The data are presented as the mean ± SEM. ^*∗∗*^*P* < 0.01, compared to the normal group; ^#^*P* < 0.05 and ^##^*P* < 0.01, compared to the model group.

AM improved the histopathological changes. Therefore, we hypothesize that AM may have a regulatory effect on DSS-induced intestinal barrier function. DSS treatment significantly reduced the expression of tight junction proteins, while the AM group exhibited increased expression of tight junction proteins in a dose-dependent manner (Figures 6(a)–6(h)). Therefore, AM-mediated protection of the intestinal barrier may be partly achieved by regulating tight junction proteins.

## 4. Discussion and Conclusion

UC is an inflammatory condition of the intestine and a vital pathogenic factor in colorectal cancer [23]. Currently, clinical treatment may involve the use of immunosuppressive agents, such as TNF inhibitors, azathioprine, and methotrexate, to suppress intestinal inflammation and control clinical symptoms [30]. However, these immunosuppressants also have potential side effects [31]. Therefore, it is important to identify effective drugs with fewer side effects for the treatment of UC.

There is evidence to support that the NLRP3 inflammasome [32] and intestinal mucosal barrier [13, 14] are related to UC. The NLRP3 inflammasome is the most widely studied cytoplasmic polyprotein complex and may be related to the production of inflammation-related cytokines. Experimental studies have confirmed that the DSS-induced ulcerative colitis model is mediated by the NLRP3 inflammasome [33]; tight junction proteins (TJP) are the basic structure of the mechanical barrier, maintain normal paracellular permeability, and prevent macromolecule passage [34]. TJPs mainly include the transmembrane proteins occludin and claudin-1 and the intracellular protein ZO-1. During the development of UC, the intestinal mucosal barrier is damaged, and the tight junctions are destroyed [13, 14]. Previous publications have reported that AM has significant protective effects against inflammatory bowel disease [17, 18]. In addition, a compound preparation (Huangkui Lianchang Decoction) also has a significant effect on UC model mice [19]. However, its mechanism has not yet been clarified or examined. Therefore, this study started with NLRP3 inflammasome and the intestinal mucosal barrier to further explore the possible mechanism of AM in the treatment of UC.

Generally, AM dose-dependently restored weight loss in DSS-induced mice. The pathological symptoms and injury in the colon were also reduced. We found that 8.2 g/kg AM significantly reduced the activity of inflammatory cytokines in the colon. In addition, AM could protect the colon against damage caused by inflammatory cell infiltration. IL-1*β* and IL-18 are proinflammatory cytokines that play important roles in intestinal inflammation. The maturation and secretion of IL-1*β*, IL-18, and caspase-1 depend on the activation of NLRP3 inflammasomes, and caspase 1 activation cleaves caspase 1 p10 and caspase 1 p20. In this study, AM inhibited the production of IL-1*β*, IL-18, and caspase 1 p10 and the protein level of NLRP3 in colon tissue, which indicates that the downregulation of NLRP3 inflammasome activation helps to alleviate DSS-induced colitis. In addition, recent studies have shown that *β*-arrestin1 is a multifunctional protein that is believed to play a role in activating the NLRP3 inflammasome [24]. Our results showed that treatment with AM could significantly inhibit the protein expression of *β*-arrestin1 in DSS-induced UC mice, suggesting that AM could downregulate *β*-arrestin1 expression and interrupt NLRP3 signaling pathway activation.

The NLRP3 inflammasome plays a vital role in maintaining homeostasis of the intestinal epithelium. Mice with NLRP3 gene defects are more likely to develop colitis in response to DSS [35]. Other studies suggest that inhibiting NLRP3 inflammasome signaling can reduce the intestinal barrier dysfunction caused by sepsis [36]. Therefore, this study examined the effect of AM on TJPs in the intestinal mucosal barrier in mice with DSS-induced colitis. The results showed that AM could increase the expression of tight junction proteins in a dose-dependent manner. Therefore, AM could alleviate the severity of colitis by regulating intestinal mucosal tight junction proteins.

In conclusion, our work showed that AM has a therapeutic effect on DSS-induced UC in mice. This therapeutic effect manifested as improvements in symptoms, reductions in inflammatory damage, the regulation of the immune response and the protection of intestinal barrier function. The underlying mechanism of these effects involves blocking the *β*-arrestin1/NLRP3 inflammasome signaling pathway and regulating intestinal tight junction proteins. However, in our current study, the correlation between AM and the NLRP3 inflammasome and intestinal mucosal barrier was still incomplete. Based on these results, AM is expected to become a potential treatment for UC and other inflammatory diseases. However, we only initially explored the effect of AM in UC mice. The pathophysiological process of UC is complex, and the protective effect and related mechanism of AM on UC remains to be further explored.

## Figures and Tables

**Table 1:** Disease activity index scores.

Score	Weight loss (%)	Stool consistency	Bloody stools
0	None	Normal	None
1	1–5	Soft and shaped	Between
2	6–10	Loose stool	Fecal occult blood
3	11–15	Between	Between
4	>15	Diarrhea	Defecate hemorrhage

DAI = (weight loss score + fecal shape score + bloody stool score)/3 [21].

**Table 2:** Histology score.

Score	Epithelium (E)	Infiltration (I)
0	Normal morphology	No infiltrate
1	Loss of goblet cells	Infiltrate around crypt basis
2	Loss of goblet cells in large areas	Infiltrate reaching to L. muscularis mucosae
3	Loss of crypts	Extensive infiltration reaching the L. muscularis mucosae and thickening of the mucosa with abundant edema
4	Loss of crypts in large areas	Infiltration of the L. submucosa

The total histological score represents the sum of the epithelium and infiltration score (total score = E + I).

**Table 3:** Primer sequences for qRT-PCR.

Primer name	Primer sequence (5′-3′)
m-IL-1*β*-F	CCAAGCTTCCTTGTGCAAGTA
m-IL-1*β*-R	AAGCCCAAAGTCCATCAGTGG
m-IL-18-F	GCATCAGGACAAAGAAAGCCG
m-IL-18-R	AGTTGTCTGATTCCAGGTCTCCAT
m-GAPDH-F	AGGAGCGAGACCCCACTAACA
m-GAPDH-R	AGGGGGGCTAAGCAGTTGGT
m-ASC-F	AGACATGGGCTTACAGGA
m-ASC-R-	CTCCCTCATCTTGTCTTGG
m-NLRP-3-F	GTGGTGACCCTCTGTGAGGT
m-NLRP-3-R	TCTTCCTGGAGCGCTTCTAA
m-Caspase 1-F	TATCCAGGAGGGAATATGTG
m-Caspase 1-R	ACAACACCACTCCTTGTTTC

## Data Availability

No data were used to support our research results.
